# Study Protocol to Determine Association between Environmental Triggers and Asthma among Children in King Williams Town

**DOI:** 10.3390/mps4030064

**Published:** 2021-09-10

**Authors:** Rasaq A. Yusuf, Phoka C. Rathebe, Wells Utembe

**Affiliations:** 1 Department of Environmental Health, Faculty of Health Sciences, Doornfontein Campus, University of Johannesburg, P.O. Box 524, Johannesburg 2006, South Africa; powerray2003@yahoo.com (R.A.Y.); WellsU@nioh.ac.za (W.U.); 2 National Health Laboratory Service, Toxicology and Biochemistry Department, National Institute for Occupational Health, Johannesburg 2000, South Africa

**Keywords:** asthma, environmental agents, children, exposures, chronic

## Abstract

Asthma affects over 330 million people worldwide, with relatively higher disease burdens in Australia, New Zealand, Africa, the Middle East, and South America. The symptoms associated with asthma were reported to be prevalent in children from the period of 1993 to 2013, in many low- and middle-income countries, due to changes in environmental conditions, such as domestic lifestyle, and urban and industrial developments. (1) Background: Several studies have also shown that children are prone to a severe type of asthma, because of their narrow respiratory airways and susceptibility to irritation from environmental agents. This study aimed to assess the association between environmental exposure and asthma among children in King Williams Town, South Africa. (2) Methodology: This study adopted a cross-sectional design method, with an estimated sample size of 262 participants. The eligible study participants were enrolled while attending Grey hospital in King Williams Town, for asthma management. Information will be collected from eligible, stable participants, on asthma treatment, through in-person interviewing in 2021. A semi-structured questionnaire will be administered to the participants. However, as a result of the prevailing COVID-19 pandemic, data may be abstracted from the asthma medical record of the eligible participants. Multivariate regression will be utilized, to describe the correlation between the variables, and the odds ratio will be calculated as well. (3) Discussion and conclusion: The study will objectively identify the local environmental agents that are associated with asthma among children in King Williams Town, in order to reprioritize treatment and preventative strategies. Ethical approval was obtained from the Research Ethics Committee, Faculty of Health Sciences at the University of Johannesburg.

## 1. Introduction

Asthma is a chronic lung disease affecting over 330 million people worldwide, with relatively higher disease burdens in Australia, New Zealand, Africa, the Middle East, and South America. Asthma symptoms have reportedly become more prevalent in children from the period of 1993 to 2013, in many low- and middle-income countries, due to changes in the environmental conditions, such as domestic lifestyle, and urban and industrial developments [1]. The pathophysiological changes that are associated with asthma are described as early and late responses. In the early response, exposure to an environmental agent, through inhalation, ingestion, or through skin contact, stimulate certain specialized inflammatory cells (mast cells) in the airway, which cause cross-linking of immunoglobulin E (Ig E) on the mast cells surface, to release histamine. Subsequently, histamine triggers the production of prostaglandins, leukotrienes, and other enzymes. In addition, cytokines, which are also derived from the mast cell, will signal other airway lining cells to release their inflammatory mediators to the lung. These interactions, which occur within minutes, result in the characteristic response of asthma—increased vascular permeability, mucus secretion, bronchospasm, wheezing, chest tightness, and cough [2,3,4]. The most significant component of the early asthma response is bronchospasm—a reversible spasmodic response of the airway smooth muscle, which leads to a narrow airway and decreased oxygenation.

The late asthmatic response is a consolidated process that occurs after hours of the initial response. It is caused by a number of cellular inflammatory processes continuing the initial response. Antigen-presenting T cells trigger T helper cells through the allergenic antigen process, causing secretion of multiple cytokines that are able to maintain and intensify the local inflammatory response. Furthermore, mast cells, eosinophils, histocytes, neutrophils, and other inflammatory cells, respond to the cytokines from antigen-presenting T cells, in order to produce more cytokines. These inflammatory cells will all produce more cytokines, to amplify the cellular response, inflammatory reaction, bronchospasm, and asthma symptoms [3,4]. Overall, the late asthmatic response is a migration of inflammatory cells from the circulation into the pulmonary vasculature and the airway submucosa.

Asthma is more severe in children because they have narrow airways that are still developing and are susceptible to irritation from environmental agents [5,6]. Such irritation may become pronounced in toddlers, who tend to crawl around, and are often exposed to dust, which is a possible asthma trigger in this age group. Older children are equally exposed dust and other environmental agents while playing. These associations may be synonymous with children in King Williams Town, of which many belong to black ethnicity and low socioeconomic status with poor nutrition, and thereby impacting cellular growth and maturation, with the consequence of influencing body vulnerability to environmental exposure. Furthermore, a narrow and hyperreactive airway, coupled with an obligate nose breathing pattern, among neonates and infants, worsen the effects of environmental exposure among children with asthma.

Wjst and Boakye described childhood asthma prevalence among some Africa countries as 9.1% in Ethiopia, 15.8% in Kenya, 20.3% in South Africa, 8.7% in Algeria, and 11.9% in Tunisia [4]. The prevalence among children was linked with endemic poverty and environmental circumstances among these Africa countries [7]. It was also identified that a general lack of resources, poor accessibility to healthcare and an inadequate number of healthcare workers, and environmental pollution, contributed to the rising trend of childhood asthma and mortality in Africa [7,8]. The outcomes of Chu et al.’s longitudinal study, involving 39,907 singleton children who were followed-up for between 1959 and 1976, showed that women who were treated for pregnancy-related infection with penicillin or chloramphenicol had increased risk of asthma in their children, with an adjusted odds ratio = 1.21, 95% confidence interval (1.08–1.36) for penicillin; and 1.72 (1.14–2.59) for chloramphenicol [9]. Essentially, in utero exposure of an unborn baby to certain medications is associated with childhood asthma. The incidence of association between food allergy and asthma in children was reported by the National Cooperative Inner-City Asthma Study (NCICAS). Five hundred and four random serum samples were evaluated for specific immunoglobin E (Ig E) to common food allergens (egg, milk, soya beans, peanut, wheat, and fish). Forty-five percent of the patients had evidence of sensitization (food-specific IgE ≥ 0.35 kU/L) to at least one of the food items. The main finding of the study was that food allergen sensitization is highly prevalent and it is associated with asthma in children, increased asthma healthcare, and medication use [10]. Although there are differences in the regional prevalence of asthma in South Africa, rural and urban prevalence rates have continued to rise since 1995, due to rapid urbanization and environmental pollution. In Buffalo City Metro Health District (BCMHD), which includes King Williams Town, a total of 171 children died from asthma and asthma-related events, between 2010 and 2015 [11]. With the ongoing concerns on environmental pollution in King Williams Town, illegal dumping, outdoor burning of refuse, and indoor use of solid fuels, these may be the nidus for asthma attacks and deaths among children within the locality.

In different parts of the world, many environmental factors have been shown to affect the incidence of asthma. For example, in Europe, environmental factors, such as irritant gases, inorganic particles, allergens, and environmental tobacco smoke, have been associated with childhood asthma [12]. On the other hand, environmental factors, such as transport-related air pollutants (TRAP), pollen dust, particulate matters, and industrial emissions, were associated with childhood asthma in the United States of America (USA) [13]. A cross-sectional study by the International Study of Asthma and Allergies in Childhood (ISAAC) assessed the correlation between environmental exposure and childhood asthma among school children aged 6–14 years in Cape Town and Polokwane, among over 6000 participants. There was significant effects of environmental agents (environmental tobacco smoke, pollen dust, TRAP, industrial emissions) on asthma prevalence among the participants [14,15]. Nevertheless, research on the association between environmental exposure and asthma, among children in King Williams Town, has not been previously conducted. Therefore, this study aimed to assess the association between environmental exposures (indoor environmental agents, outdoor environmental agents, allergenic foods, and certain drugs) and asthma among children who are between the age of 3 and 12-years, in King Williams Town. Some of the agents to be considered in this study are as follows: indoor (environmental tobacco smoke, cooking smoke); outdoor (transport-related air pollutant, dust, pollen dust); allergenic foods (egg, milk, seafoods, pulses); certain medications (panado, penicillin, metronidazole); and physical exercise. The scope of this study will not involve eliciting underlying medical conditions, medical investigations, such as the lung function test, skin prick test, or measurement of the blood oxygen saturation level. Essentially, the following objectives will be pursued to achieve the aim of this study:

- To identify environmental triggers that are linked with asthma among children who attended Grey hospital, King Williams Town, between January and December 2021;
- To measure the frequency of exposure to different environmental triggers of asthma;
- To describe the impact and severity of environmental agents on asthma symptoms among participants, by age, gender, and racial groups.

## 2. Methodology

The proposed study will adopt a descriptive, cross-sectional design to assess the association between environmental triggers and asthma among children who are 3–12 years old in King Williams Town. The Sp0_2_ 95% would be required to participate. This study will commence with data collection from January 2021 and is anticipated to end November 2021.

### 2.1. Study Setting

This study will be conducted in King Williams Town, Eastern Cape; a commercial center within the southeastern part of South Africa, at an altitude of 405 m above sea level, −32.8808 latitude and 27.3945 longitude. The climate is classified as warm and temperate, with an average annual temperature of 18 °C and average annual rainfall of 600 mm [16]. The town is often dusty during the summer season, due to the dust emanating from the surrounding untarred roads as well as pollen dust during autumn.

### 2.2. Study Population and Sample Size

The total population of King Williams Town, with the surrounding locations, is 227,000 with an estimated child (3–12 years old) population of 54,000 [17]. This is in reference to the population of children in King Williams Town, a town situated in Buffalo City Metro Municipality, Eastern Cape. The target population will be comprised of children managed for asthma, either symptomatic or asymptomatic, but stable on asthma medication. Participants will be selected among the children who attended the hospital for asthma treatment during the study period. The sample size was determined using Epi Info. 7.20, with 95% confidence interval, 80% statistical power, ratio of 2:1, 25% as the percentage of outcome in the unexposed group, 1.7 risk ratio, and 2.43 odds ratio [18], with 51.0% as the percentage of outcome in the exposed group; the estimated sample size (Fleiss with continuity correction factor) = 209. The assumptions of outcomes in the unexposed and exposed groups were based on a literature review [18], which is needed to input in the Centers for Disease Control and Prevention (CDC) Epi Info software. Furthermore, an additional 25% contingency, which is 52.25, approximated to 53, was added. Therefore, the sample size for the proposed study is 209 + 53 = 262. This study seeks to include children who are 3–12 years old and attended Grey hospital for asthma management with their caregiver/parent or guardian, between January and November 2021. Children will be divided into the following three age groups: 3–5; 6–8; and 9–12. However, children who are managed for asthma and are older than 12 years, reside outside King Williams Town, and are treated for non-asthmatic medical condition(s), will not be invited to participate. Grey hospital, a district hospital, is not sophisticated enough to manage children younger than 3 years. Hence, children younger than 3 years old were excluded.

### 2.3. Data Collection

#### 2.3.1. Semi-Structured Questionnaires

Due to the vulnerability of children as the subjects of this research, caregivers/parents/guardians of the eligible children will only be interviewed and respond to questions on behalf of their child/children, during admission in the emergency room, in the wards during hospital admission, or at follow-up in the outpatient clinic in doctor consultation rooms, based on the ISAAC questionnaire [19]. The questionnaire will be semi-structured and administered with the help of two trained support staff and is to be completed between 30 min and 1 h. The questionnaire is attached. All the eligible participants will be selected while attending Grey hospital, King Williams Town for asthma management during data collection phase. Children with their respective caregivers/parents/guardians will be allowed in doctor consultation rooms where research interactions and conversations cannot be heard by others. Only caregivers/parents or guardians of eligible children will be interviewed and respond to questions on behalf of their child/children. However, the COVID-19 etiquettes (handwashing, social distancing, and use of facemask) will be observed throughout the study period. Participants with respiratory symptoms, with COVID-19 diagnosis, or who are too ill to participate, will be excluded from the study.

#### 2.3.2. Asthma Records Review

Due to the ongoing COVID-19 pandemic, participants were not available for in-person interviews during the pilot study and the actual study. As of the time of writing this manuscript, South Africa is currently on level 3 movement restrictions, another impediment to gather primary data. Therefore, the ISAAC questionnaire, which was originally designed for gathering primary data, was modified for data abstraction, reflecting the core components described by Banks [20]. A copy of the data abstraction is attached. Our approach with the use of secondary data helps to save time, resources, and prevent exposure and transmission of coronavirus. In addition, the new approach will strengthen our problem-solving skills to answer research questions, nurture a conceptual model testing the use of a new statistical method for data collection during a pandemic and times when access to primary data becomes difficult. Essentially, the pandemic has made the researchers consider a novelty approach for when the collection of primary data becomes problematic. In this study, records of environmental exposures that were recorded in each patient medical record are being abstracted to suit the aim and objectives of this study, starting from January 2021. The secondary data access request was granted by the Higher Degrees Committee at the University of Johannesburg (MPH HDC-01-61-2020).

### 2.4. Sampling

In this study, a descriptive, cross-sectional study design is adopted with a simple random sampling technique. Participants were selected by taking into context the study period between January and November 2021. All the eligible participants recruited into the study was based on inclusion and exclusion criteria, to obtain the required sample size of 262.

### 2.5. Reliability and Validity

The format of the questionnaire and options in the questions are based on the precept of ISAAC—an international organization with the largest worldwide collaborative childhood asthma research in 105 countries, involving over 2 million children [14]. According to Eckerdal and Hagstrom, a standardized questionnaire from a reputable scientific body could be used as the basis for the new research [21]. Responses to the modified questionnaire were compared among interviews conducted by the researcher and the two support staff for reproducibility of the results. For instances where there are discrepancies, these will be corrected during the initial pilot study. In a situation where there was language barrier, support staff assisted in translating questions in the dominant language spoken in Eastern Cape (isiXhosa).

Face validity was utilized to test whether the modified questionnaire was designed to measure the concept that was being tested. This was accessed by conducting a pilot study involving 10 participants to evaluate if the questions are relevant, clear, and unambiguous, and adjustments were made where necessary. The aspect of content validity of the questionnaire was tested against ISAAC questionnaire format, in order to know whether enough and relevant questions were outlined in the study questionnaire. In order to prevent misrepresentation and mistakes by the responders, the two support staff will be available throughout data collection phase to translate questions where necessary, into isiXhosa. Due to the ongoing pandemic, the 10 participants with their carers did not show up and the interviews were cancelled. In lieu of this, a pilot study was performed with data abstraction from patients medical records.

### 2.6. Data Analysis

Data collected will be entered into IBM^®^ SPSS software version 26.0 for statistical analysis. In order to describe the population, absolute and relative frequencies will be calculated. Also, crude and adjusted odd ratios (OR) as well as 95% confidence interval (CI) will be calculated to evaluate asthma among the children and the identified environmental agents. Adjustments will be made for variables that are statistically significant *p*-value ≤ 0.05.

#### Analysis by Objective

Objective 1: Crude ratio, adjusted odds ratios (OR) and 95% confidence intervals (CI) will be calculated to estimate the likelihood of participants developing childhood asthma due to the presence of environmental exposure.

Objective 2: Frequency of exposure to different environmental agents and asthma among the participants will be explored using frequency distribution table.

Objective 3: The significances of dependent variable (childhood asthma) and independent variables (environmental agents) will be analyzed with multivariate regression, with stratification of outcomes according to age, gender, and racial group.

## 3. Ethics and Dissemination

This study was granted ethical clearance (REC-783-2020) for non-therapeutic research involving minors, from the Faculty of Health Sciences Research Ethics Committee (REC) of the University of Johannesburg in 2020. Furthermore, a secondary data access request has been granted by the Higher Degrees Committee at the University of Johannesburg (MPH HDC-01-61-2020), and ethical clearance has been noted by the Eastern Cape Provincial government. Consent was sought from the study participants after understanding that their participation in the study will be voluntary and may they choose to withdraw from the study at any time, without any repercussion. The research participants’ right to privacy was respected and the researcher will, at all times, ensure that all the information of the participants is securely stored, used, and subsequently destroyed, to protect the participant’s identities. The eligible children for the study, with their caregivers/parents or guardians, were allowed in the doctor consultation rooms, where the research interactions and conversations could not be heard by others. Confidentiality was maintained through the use of unique identification numbers instead of using participants names on the questionnaire. All the information retrieved from the study will be kept private, confidential, and anonymous. No personal details of any participants will be published. After collecting the data, the questionnaires will be kept in locked cabinets and will only be accessible by the researcher. The collected data, in electronic format, will be stored on a password-protected computer and retained for the duration of years, as required by the University of Johannesburg Research Ethics Committee.

**Figure 1:**
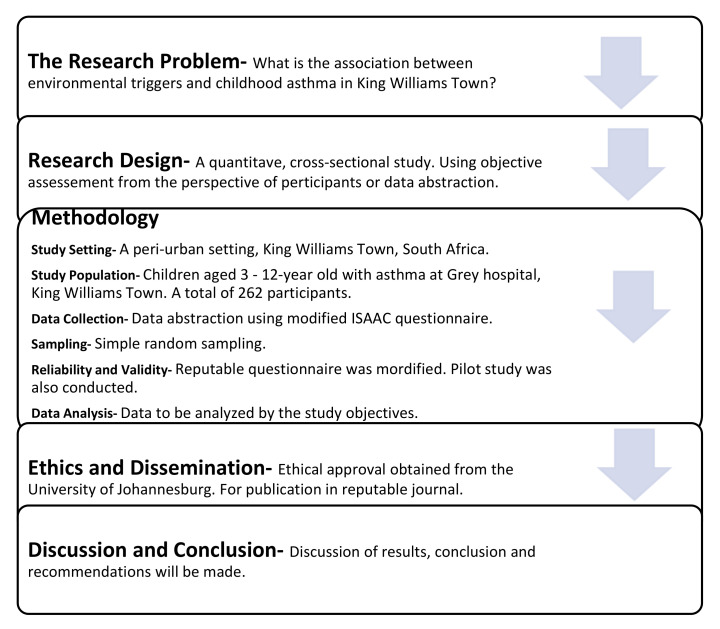
A flow diagram of the manuscript.

Upon completion of this study, the children and caregivers in King Williams Town, South Africa, will be able to identify and avoid environmental agent(s) that could trigger pediatric asthma. Healthcare workers at Grey hospital will be able to objectively identify common environmental agents that are associated with asthma among children. Asthma treatment within the locality will be reprioritized, to focus on environmental factors as well as health education. A flow diagram of this manuscript, identifying the various stages, is provided in Figure 1. 

## 4. Discussion

The living standard of people in rural areas is often affected by the environmental situation, poor resource allocation, prevailing poverty, and socioeconomic inequalities. These situations interact as a prelude to how people are exposed to certain environmental agents. King Williams Town is a rural settlement, with a mix of peri-urban scenarios, where an average household still uses wood as a source of indoor heating and cooking. Solis-Soto et al., in a rural village in Bolivia—a developing country with comparable environmental scenarios to South Africa—showed that, among school children with a median age of 11 years, indoor air pollution from cooking smoke was associated with asthma and allergies [22]. While comparing the children with their fellow urban residents, the children living in rural areas were more exposed to environmental tobacco smoke (ETS), smoke from wood or coal as cooking fuel, and farm animals. In contrast, urban residents are often more exposed to traffic-related air pollution (TRAP) and industrial emissions. The uniqueness of King Williams Town, as a commercial nerve center in the Amathole district, with sprawling industrial developments, predisposes the children to environmental pollutants. Consequentially, the common environmental agents that are likely to be associated with asthma among children, in both rural and urban areas, are invariably present in King Williams Town. The importance of this study will be hinged on unpacking these environmental agents with objective evaluation.

South Africa has four seasonal weather patterns—summer, autumn, spring, and winter. During winter, the lower environmental temperature encourages rapid multiplication of viral particles, leading to the prevalence of influenza and respiratory diseases. A retrospective study, to evaluate the relationship between winter respiratory syncytial viral infection and the development of asthma among nearly 100,000 infants, showed that children who are 4 months of age, at the peak of the winter viral season, are more at risk of developing both clinical bronchiolitis and childhood asthma [23]. In the context of King Williams Town, the children whose parents cannot afford an air conditional heater are continuously at risk of recurrent asthma attacks, due to the developmental nature of their airway, endemic respiratory viral infections, weather pattern, and seasonal variation. It is anticipated that caregivers would be able to provide their experience of the relationship between weather patterns and asthma in their children.

Despite the on-going improvement strategies embarked on by the government, to improve the socioeconomic condition of the people in Eastern Cape and other provinces in South Africa, many people still live in informal settlements. Dwelling in informal houses (shacks) with leaking roofs and poor ventilation systems, will likely predispose local residents to high indoor humidity and the risk of asthma in children. Also, with the prevailing high level of unemployment in South Africa, this could imply that the little available household cash is spent on alcohol and cigarettes. Hallit and Salameh found a significant association between asthma and the children of mothers who engaged in waterpipe smoking during pregnancy, in a cross-sectional study of a cohort of 527 children (*p* value= 0.003; OR = 13.25; 95% CI 2.472–71.026) [23]. It was reiterated that the use of waterpipe smoking during pregnancy, alcohol consumption, and living in houses with poor infrastructures, are associated with a higher possibility of childhood asthma. Cases of pregnant women who smoked cigarettes and consumed alcohol during pregnancy are often reported to nurses in maternity centers around King Williams Town. A formal assessment into this assertion has not been previously conducted in King Williams Town. Some of the questions in the questionnaire of this study are focused on tackling smoking during pregnancy.

## 5. Conclusions

The design of the questionnaire was broad-based, with some open-ended questions; these will allow the researcher to elicit certain pertinent issues on asthma that matter most to participants, through data abstraction. The study will help to unpack the underlining values and assumptions among the children and their caregivers/parents/guardians, in order to gain an understanding of the driving forces contributing to environmental triggers and childhood asthma within the King Williams Town. The opportunity to undertake this study will allow for the exploration of views and perspectives of children who are from different racial backgrounds.

The centrality of this study is the reliance on the information abstracted on environmental exposure and childhood asthma. Therefore, there is a tendency for attrition of record, and incomplete records cannot be ruled out. However, in order to make-up for missing or incomplete records, more data will be collected, more than the required sample size, and up to 400 data samples. There are non-environmental factors that may trigger childhood asthma, such as stress, phobia, obesity, and genetic predisposition; this study could not keep track of all these factors.

## Data Availability

Not applicable.
